# Prevalence and antimicrobial resistance of extended spectrum beta-lactamase (ESBL) producing *Klebsiella* spp. in poultry meat

**DOI:** 10.1016/j.heliyon.2025.e41748

**Published:** 2025-01-06

**Authors:** Fatema Yeasmin Tanni, Md. Shahidur Rahman Chowdhury, Hemayet Hossain, Md. Atik Faysal, Md. Anisur Rahman, Ahsan Al Emon, Mashuka Nahida Asha, Md. Mukter Hossain, Md. Mahfujur Rahman

**Affiliations:** a Department of Medicine, Sylhet Agricultural University, Sylhet, 3100, Bangladesh; b Department of Biochemistry, University of Nebraska-Lincoln, Beadle Center, 1901 Vine St, Lincoln, NE, 68503, United States; c Department of Anatomy and Histology, Sylhet Agricultural University, Sylhet, 3100, Bangladesh; d School of Veterinary Medicine and Biomedical Sciences, University of Nebraska-Lincoln, NE, 68583, United States

**Keywords:** Prevalence, Antimicrobial resistance, ESBL, *Klebsiella* spp., Poultry meat

## Abstract

*Klebsiella* spp. present in the food chain have been of much interest during the last few decades due to their implication in the development of antimicrobial resistance. We determined the prevalence of *Klebsiella* spp. (*Klebsiella pneumoniae* and *Klebsiella oxytoca*) in retail poultry meat, along with their resistance profile against antimicrobials. For the detection of the *Klebsiella* spp. a total of 285 raw meat samples of retailed poultry were collected and cultured. All positive cultures were screened for *Klebsiella* spp. by PCR to confirm the identity. Their antimicrobial susceptibility pattern was performed using the disk diffusion technique, whereas the ESBL-coding genes were detected using the multiplex PCR technique. Out of 285 samples, 139 (48.77 %) raw meat samples had *Klebsiella* spp. While out of 139 identified positive isolates, 99 (34.74 %) isolates were *K. pneumoniae*, and 13 (4.56 %) were *K. oxytoca*. Phenotypically, most of the strains were fully (100 %) resistant to ampicillin, amoxicillin, cefuroxime, ceftazidime, and nalidixic acid. Of the β-Lactamase genes that were detected through mPCR, *bla*_*SHV*_ was the dominant gene among the strains *K. pneumoniae* and *K. oxytoca* isolates, with percentages of 86.87 % and 76.92 %, respectively. Besides, isolates of *K. pneumoniae* harbored 95.96 % (95 % CI: 89.98–98.89) of the *tetA* gene, whereas the *K. oxytoca* isolates harbored only 92.31 % (95 % CI: 63.97–99.81) of the *tetA* gene. Most of the *K. pneumoniae* and *K. oxytoca* isolates were found to harbor the streptomycin-resistant gene, *strA*. These results highlighted significant carriage of multidrug-resistant (MDR) *Klebsiella* spp. in retail poultry meat, which insinuates that there is a need for a strategic plan in place to curb the dissemination of these multi drug resistant pathogens along the food chain.

## Highlights

• High prevalence of *Klebsiella pneumoniae* (34.74 %) and *Klebsiella oxytoca* (4.56 %) was detected in retail poultry meat.
• *Bla*_SHV_ was the most dominant ESBL gene, found in 86.87 % of *K. pneumoniae* and 76.92 % of *K. oxytoca* isolates.
• All isolates showed multidrug resistance (MDR), with MAR index values indicating exposure to highly resistant environments.
• Meropenem, gentamicin, and amikacin showed high sensitivity, while β-lactam and quinolone resistance were ubiquitous.

## 1 Introduction

The worrisome global increase of antibiotic resistance has come to be acknowledged as a persistent public health concern in recent years [1]. According to projections, antimicrobial resistance (AMR) will surpass all other causes of death worldwide by 2050, with an estimated 10 million deaths per year, up from 1.2 million in 2019 [2]. It has become a critical global public health challenge, affecting both developed and developing nations. The critical implications of AMR for medical clinical practice have mainly concerned patients with serious infections who required ICU hospitalization. *Klebsiella pneumoniae* is one of the Gram-negative bacteria of particular concern because of its capability for the rapid acquisition and further spread of resistance mechanisms [3]. In developed countries, AMR in *K. pneumoniae* is a growing issue, as highlighted in studies showing its high prevalence and resistance to critical antibiotics like carbapenems and cephalosporins in healthcare settings [4]. Similarly, in developing countries of Asia, including Bangladesh, AMR in *K. pneumoniae* presents a severe healthcare burden as healthcare-associated Infections (HAI), with high resistance rates often complicating treatment outcomes in clinical settings [5].

The poultry sector holds significant importance within agricultural systems, fostering employment opportunities, enhancing food security, and augmenting access to quality protein in dietary landscape of Bangladesh [6]. According to the DLS & BPICC report 2019, the country's daily production of poultry products saw a substantial rise [7]. In spite of the growth of this industry, it is also burdened with the problem brought about by the practice of adding antibiotics routinely to poultry feed at sub-therapeutic levels to enhance growth, alongside their use for therapy and disease prevention [8]. Antibiotics taken globally are used more than 50 % in food animals, and by the year 2030 a projected growth of 50 % rise in the use of antibiotics in farming [9,10]. World Health Organization has identified antibiotic-resistant bacteria globally as a serious public health concern of the twenty-first century, may emerge and spread as a result of the incessant application of antibiotics in poultry farming to foster growth and avert diseases [11,12]. The abundance of organisms responsible for producing ESBL is the primary trigger of bacterial infections that exhibit elevated antibiotic resistance [13].

Penicillin, aztreonam and cephalosporins can all be hydrolyzed by the ESBL enzyme, and clavulanic acid, a beta-lactamase inhibitors can reduce this enzyme's activity [14]. All kinds of commercial hens, including recently hatched ones, encompass ESBL-producing bacteria. Gram-negative bacteria, especially those in the Enterobacteriaceae family, are the main source of this enzyme. TEM-52, SHV-12, and CTX-M-1 are the most common ESBL types encountered in poultry and poultry products [15].

*K. pneumoniae* is a prime etiology of pulmonary infection that causes a high death rate in broilers and chicks [16,17]. The occurrence of *K. pneumoniae* was documented at a rate of 10 % among specimens collected from chickens exhibiting respiratory illnesses in Egypt [18]. Furthermore, a recent investigation has illustrated the presence and endurance of *K. pneumoniae* within poultry slaughterhouse surroundings, potentially facilitating its dissemination throughout the poultry meat production chain [19]. Furthermore, effluent from pig and chicken slaughterhouses has recently come to light as a common source of multidrug-resistant (MDR) *K. pneumoniae* [20]. Poultry and meat vendors face disease exposure during slaughtering, processing, and selling, particularly in low-income countries where working environments are often unhygienic and hazardous [21,22]. In the battle against antimicrobial resistance, the correlation between chicken meat consumption and overall human health is emerging as a critical concern within the poultry sector, particularly regarding food safety [23]. Among the clinically infected patients in Bangladesh, *K. pneumoniae* presence in 44.22 % of human infections, with strains demonstrating notably high resistance rates to most commonly used members of penicillin, cephalosporin and beta lactamase inhibitor groups [24].

To date, limited studies have been conducted on isolating *Klebsiella* spp. from livestock samples in Bangladesh [25]. However, molecular detection of *Klebsiella* spp. from livestock products in Bangladesh has not been documented thus far. With the escalating demand for broiler meat in Bangladesh, similar to other countries, there is a pressing need to ascertain the prevalence of ESBL-producing *Klebsiella* spp. in chicken meat with characterization of antimicrobial resistance profiles. So, the study sought to determine the prevalence of *Klebsiella* spp. (*K. pneumoniae* and *K. oxytoca*) in retail poultry meat and to assess their antimicrobial resistance profiles.

## 2 Materials and methods

### Ethical Statement

Both the study protocol and the poultry meat examinations were approved under the guidelines of the Animal Experimentation and Ethics Committee (AEEC) at Sylhet Agricultural University, Bangladesh (#AUP2022039).

### 2.1 *Researchdesignandsamplingplan*

**Fig. 1:**
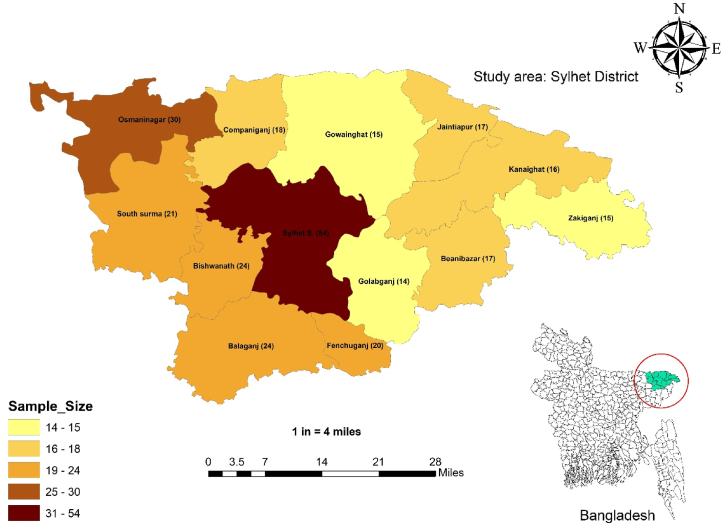
The map showing the study area (Sylhet district) with specific sample size in different upazilas.

From January to March 2023, a cross-sectional study was conducted across 13 upazilas in Sylhet, Bangladesh. As illustrated in Fig. 1, these regions are geographically located between 24°36′ and 25°11′ Northern Hemisphere Latitude and 91°38′ and 92°30′ Eastern Hemisphere Longitude. Accessible sampling was used to collect samples, with an emphasis on retailer chicken meat outlets. In order to accurately estimate the prevalence, the minimal sample size was assessed using the formula as follows [26,27]:

Equation (1):

(1) $$n=\frac{Z^{2}Pexp\left(1-Pexp\right)}{d^{2}}$$

Where, Pexp = expected prevalence, and d = required absolute precision, z = 1.96 for the 95 %

confidence interval (CI) level. A prevalence rate of 16 % (Pexp = 0.16), as determined by a previous meta-analysis in Nepal, was used to refine the sample size calculation [28]. The minimal sample size was 207 with an absolute precision of d = 0.05. In order to find *Klebsiella* spp. that encode ESBL, 285 samples of chicken meat were examined in the current study.

### 2.2 *Collectionofsamplesandisolationofbacteria*

A total number of 285 chicken meat swab samples were gathered from various retail outlets in the Sylhet district. In order to preserve the proper temperature during transportation, swab samples from chicken meat were aseptically collected in disinfected containers containing buffered peptone water (BPW; Oxoid, UK) and brought to the lab in a refrigerated box. At first each sample was inoculated into BPW and underwent incubation at 37 °C for 24 h as pre-enrichment. The increase of turbidity in the BPW media was a sign of bacterial proliferation. The enriched samples were cultured on Eosin Methylene Blue (EMB; Oxoid, UK) agar media following pre-enrichment, and then they were proceeded with aerobic incubation for 18–24 h at 37 °C. After sub culture into MacConkey agar (Oxoid, UK) plates, the suspected colony underwent incubation for 24 h at 37 °C additionally. The initial screening for probable *Klebsiella* spp. was made easier by the characteristic colony morphology, notably the pink colonies that were indicative of lactose fermentation. Biochemical assays were performed after selection to verify the isolates' identities. The isolates' capacity to ferment lactose was confirmed by the lactose fermentation test conducted on MacConkey agar. Additionally, the ability to hydrolyze urea was also evaluated using a urease test, which showed a pinkening of the medium. The isolates' ability to use citrate as the exclusive carbon source was tested by the citrate utilization test, which again showed a color shift. The methyl red-Voges Proskauer (MR-VP) test helped characterize the isolates by distinguishing between 2,3-butanediol fermentation and mixed acid fermentation, while the indole test assessed tryptophan production, as shown by a color shift with the inclusion of Kovac's reagent. Finally, a motility test was used to assess whether the isolates were motile or not, which is a crucial trait for *Klebsiella* spp.. All tests were conducted following standard microbiological protocols and the guidelines followed by Liza et al. [3]. Lastly, PCR was performed for final confirmation on the isolates of *Klebsiella* spp. that were tentatively positive.

### *2.3* *Identification of Klebsiella* spp. *by PCR*

A commercial extraction kit (AddBio Inc. Ltd., Daejeon, Korea) was utilized for the extraction of genomic DNA from the *Klebsiella* isolates. For the detection of *K. pneumoniae* and the *Klebsiella* genus, multiplex PCR was employed while uniplex PCR was utilized for *K. oxytoca*. The primer sequences utilized are presented in Table 1, and the specific thermal cycling parameters, durations, and requirements for the PCR process are detailed in Supplementary Tables 1–2. During gel electrophoresis, positive and negative controls were added to ensure accuracy. For quality control, the reference strain *Pseudomonas aeruginosa* ATCC 27853 was employed in the study.

**Table 1:** Primer designing for identification of ESBL resistance *Klebsiella* spp., *K. pneumoniae and K. oxytoca* isolated from poultry meat.

Type of PCR	Primers (Gene)	Targeted Genes/Organism	Primer sequences	Amplicon size (bp)	Reference
mPCR-I	*gyr A*	*Klebsiella* spp.	F-CGCGTACTATACGCCATGAACGTA	441	[66]
			R-ACCGTTGATCACTTCGGTCAGG		
	*rpo B*	*K. pneumoniae*	F-CAACGGTGTGGTTACTGACG	108	
			R-TCTACGAAGTGGCCGTTTTC		
UniPCR	*peh X*	*K. oxytoca*	F-GATACGGAGTATGCCTTTACGGTG	343	
			R-TAGCCTTTATCAAGCGGATACTGG		
mPCR-II	*bla*_TEM_	*TEM-1 & 2*	F-CATTTCCGTGTCGCCCTTATTC	800	[33]
			R-CGTTCATCCATAGTTGCCTGAC		
	*bla*_SHV_	*SHV-1*	F-AGCCGCTTGAGCAAATTAAAC	713	
			R-ATCCCGCAGATAAATCACCAC		
	*bla*_OXA_	*OXA-1,4 & 30*	F-GGCACCAGATTCAACTTTCAAG	564	
			R-GACCCCAAGTTTCCTGTAAGTG		
	*bla*_CTX-M1_	*CTX-M-1, CTX-M-3, & CTX-M-15*	F-TTAGGAAATGTGCCGCTGTA	688	
			R-CGATATCGTTGGTGGTACCAT		
	*bla*_CTX-M2_	*CTX-M-2*	F-CGTTAACGGCACGATGAC	404	
			R-CGATATCGTTGGTGGTACCAT		
	*bla*_*CTX-M9*_	*CTX-M-9 & CTX-M-14*	F-TCAAGCCTGCCGATCTGGT	561	
			R-TGATTCTCGCCGCTGAAG		
	MultiCase ACC	*ACC-1 & ACC-2*	F-CACCTCCAGCGACTTGTTAC	346	
			R-GTTAGCCAGCATCACGATCC		
	*MultiCase* MOX	*MOX-1, MOX-2, CMY-1, CMY-8 to CMY-11& CMY-19*	F-GCAACAACGACAATCCATCCT	895	
			R-GGGATAGGCGTAACTCTCCCAA		
	*MultiCase* DHA	*DHA-1 & DHA-2*	F-TGATGGCACAGCAGGATATTC	997	
			R-GCTTTGACTCTTTCGGTATTCG		
mPCR-III	*tet(A)*	Tetracycline	F-GGCGGTCTTCTTCATCATGC	502	[67]
			R-CGGCAGGCAGAGCAAGTAGA		
	*str(A)*	Streptomycin	F-ATGGTGGACCCTAAAACTCT	893	
			R-CGTCTAGGATCGAGACAAAG		
UniPCR	*aac(3)-iv*	Gentamicin	F-AGTTGACCCAGGGCTGTCGC	333	[68]
			R-GTG TGC TGC TGG TCC ACA GC		
UniPCR	*Sul1*	Sulfonamide	F-CGGCGTGGGCTACCTGAACG	433	[69]
			R-GCCGATCGCGTGAAGTTCCG		

### 2.4 *Antimicrobialsusceptibilitytesting*

The Kirby-Bauer disk diffusion method was utilized for the antimicrobial susceptibility testing of the isolates [29,30]. Each isolate was inoculated with a bacterial turbidity equivalent to 0.5 McFarland thresholds and the bacterial suspension was straightway streaked on Muller Hinton Agar (MHA) plate through a sterilized cotton swab. The preparation of 0.5 McFarland standard was followed by Hoque et al. [31]. After applying antibiotic discs within 15 min of inoculation, the plates undergo aerobic incubation conditions at 35 ± 2 °C for 16–18 h. Following incubation, the diameter of the zone of inhibition was measured and interpreted based on the CLSI guidelines. Ten (10) antimicrobial classes consisting of 18 antimicrobial agents, such as Penicillins: amoxicillin (AMX, 10 μg), ampicillin (AMP, 10 μg); Aminoglycosides: amikacin (AK, 30 μg), gentamicin (GEN, 10 μg) and streptomycin (S,10 μg); Cephems (parenteral): cefuroxime (CXM, 30 μg), cefotaxime (CTX, 30 μg), ceftriaxone (CTR, 30 μg), ceftazidime (CAZ, 30 μg); Penems: imipenem (IMP, 10 μg), meropenem (MEM, 10 μg); Tetracyclines: tetracycline (TE, 30 μg); Fluoroquinolones: ciprofloxacin (CIP, 5 μg); Lipopeptides: nalidixic Acid (NA, 30 μg); Macrolides: azithromycin (AZM, 30 μg); Folate pathway antagonists: trimethoprim-sulfamethoxazole (COT, 1.25/23.75 μg); Phenicols: chloramphenicol (C, 30 μg), were used in the assay. Antibiotics were selected following a thorough survey of more than 100 prescriptions concerning *Klebsiella* issues in poultry farms, combined with discussions with 100 poultry practitioners to determine the most frequently used antibiotics in farms affected by *Klebsiella* and also the guidelines of CLSI and The United States Food and Drug Administration (FDA).

### 2.5 *Double disk synergy test*

To identify ESBL production in *K. pneumoniae* and *K. oxytoca* isolates phenotypically, the double disk synergy test (DDST) was employed, following the methodology described by Liza et al. [3].

The process proceeded with the preparation of bacterial suspensions that were standardized to 0.5 McFarland using approaches that Hoque et al. [31] had previously described. Sterile cotton swabs were used to distribute these fluids uniformly throughout MHA plates. A disk of 30 μg amoxicillin-clavulanic acid was positioned in the centre of the agar surface. The disks of ceftriaxone (30 μg) and cefotaxime (30 μg) were then positioned 20 mm apart (center to center) from the amoxicillin-clavulanic acid disk. The plates underwent incubation for 24 h at 37 °C. After incubation, "keyhole" or synergy effect, which is an indicative sign of ESBL production, appeared. An expanded inhibition zone between the amoxicillin-clavulanate and cephalosporin disks was seen, suggesting that clavulanate restored the activity of the cephalosporin.

### 2.6 *Genomicidentificationofantibiotic-resistantgenes(ARGs)andESBLencoding genes*

Genomic identification of ARGs and ESBL-encoding genes was conducted using multiplex PCR, following the procedure delineated by Liza et al. [3]. The PCR conditions for the amplification of selected antimicrobial resistance genes included a primary denaturation for 15 min at 94 °C, 30 cycles of denaturation for 1 min at 94 °C, 1 min annealing at 63 °C, and extension for 1 min at 72 °C. A final extension was performed for 10 min at 72 °C, as stated by Siddiky et al. [32].

To detect β-lactamase genes, including *bla*_TEM,_ *bla*_SHV,_ *bla*_OXA_ *bla*_CTX-M1,_ *bla*_CTX-M2,_ *bla*_CTX-M9,_ *MultiCase*_ACC,_ *MultiCase*_MOX,_ *MultiCase*_DHA,_ PCR screening was conducted on the isolates of *K. pneumoniae* and *K. oxytoca*. The procedure involved the use of specific primers, outlined in Table 1, to achieve focused amplification of the desired genes [33]. The PCR conditions included an initial denaturation for 5 min at 95 °C, 30 cycles of denaturation for 40 s at 94 °C, annealing for 40 s at 60 °C, and extension at 72 °C for 1 min. A final extension was performed for 7 min at 72 °C, as described by Bobbadi et al. [34].

### 2.7 *Antibiogramprofiling*

The multiple antibiotic resistance index was computed as per the guidelines set by Naser et al. [27] using the formula.

Equation (2):

(2) MAR=(Thenumberofantibioticstowhichanisolatewasresistant)/(Thetotalnumberofantibioticstested).

An isolate was considered MDR when it demonstrated resistance to ≥3 different categories of antimicrobials. According to Magiorakos et al. [35], Extensively Drug-Resistant (XDR) and Pan-Drug-Resistant (PDR) definitions are as follows: XDR: An isolate is considered XDR if it shows non-susceptibility to at least one agent in all but two antimicrobial categories, leaving it susceptible to only one or two. PDR: PDR denotes non-susceptibility to every agent in every antimicrobial category.

The multiple antibiotic resistance (MAR) index varied between 0 and 1, with values near zero reflecting high sensitivity and values close to 1 representing extreme resistance. An index value of 0.20 or higher was regarded as an indicator of potential high-risk of contamination or substantial resistance.

### 2.8 *Statisticalanalysis*

The assembled data were methodically organized in Excel spreadsheets. Prevalence rates were computed based on standard formulas. For univariate analysis to investigate associations among the variables, the Chi-square test was employed. Fisher's Exact Test was performed in cases where the expected count in a cell was less than five (5) and appeared to be of at least 20 % of the cells [36]. With statistical significance set below 0.05, confidence intervals were precisely computed using the binomial distribution followed by Mahen et al. [37]. SPSS version 26 was used for data analysis. R 4.3.2 was used to estimate the genotypic and phenotypic correlations of antimicrobials using the Pearson correlation coefficient. To create a correlation plot, this study employed the “Metan” package followed by Farabi et al. [38].

### 2.9 *Geo-spatialmappingandplotting*

To map the study area ArcMap 10.8 (Esri USA), and a shapefile from (www.diva-gis.org) was utilized. Using this dataset, choropleth maps were created that successfully illustrated the prevalence of a few explanatory variables. Additionally, Origin-Pro (www.originlab.com) was utilized to illustrate the isolates' patterns of antibiotic resistance through the Polar Heat Map file exchange protocol. To generate a graph revealing the frequency of genes that encode ESBL GraphPad Prism 8.4 was used.

## 3 Results

### *3.1* *Prevalence and distribution of Klebsiella* spp.

**Fig. 2:**
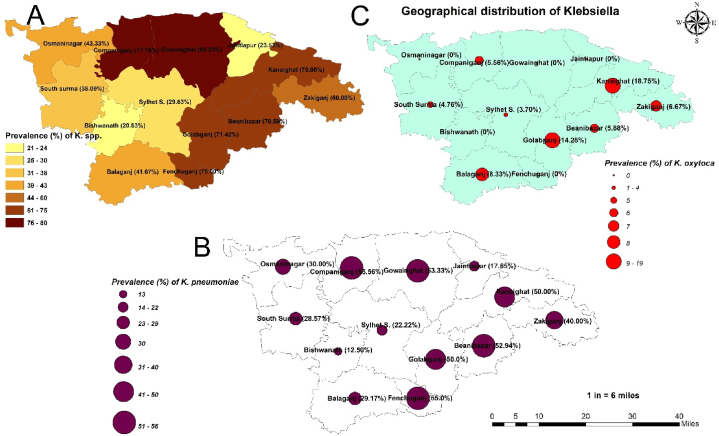
The choropleth map showing the prevalence of *Klebsiella* spp. (Fig. A) and the proportionate symbol map showing the prevalence of *K. pneumoniae* and *K. oxytoca* respectively (Fig. B & C).

Retail poultry meat in various upazilas of Sylhet district, Bangladesh, frequently harbors *Klebsiella* spp., particularly *K. pneumoniae* and *K. oxytoca*, at considerable rates (Fig. 2). From 285 samples, *Klebsiella* spp. were detected in 165 isolates, with a sensitivity of 84.24 % and a true prevalence of 57.89 %. Among the *Klebsiella* spp. positive isolates, 99 were identified as *K. pneumoniae*, with a true prevalence of 41.75 % (95 % CI: 34.41–49.68). In contrast, *K. oxytoca* was found at a significantly (*p* < 0.05) lower prevalence of 7.02 % (95 % CI: 3.36–12.99). The overall sensitivity of culture-biochemical screening compared to PCR confirmatory tests was 83.19 % for *K. pneumoniae* and 65.0 % for *K. oxytoca* (Table 2). The biochemical test results were portrayed in Supplementary Table 3.

**Table 2:** Prevalence (%) of genus and species-specific *Klebsiella* isolated from raw chicken meat samples (N = 285) from Sylhet district.

Organisms' species	Validation study (C & B)	Confirmation study (PCR)	Sensitivity (%)	Specificity (%)	Raw Prevalence (95 % CI)	True Prevalence (95 % CI)
*Klebsiella* spp.	165	139	84.24 (139/165)	100.00 %	48.77 % (42.83–54.74)	57.89 % (50.12–66.12)
*K. pneumoniae*	119	99	83.19 (99/119)	100.00 %	34.74 % (29.22–40.58)	41.75 % (34.41–49.68)
*K. oxytoca*	20	13	65.00 (13/20)	100.00 %	4.56 % (2.45–7.67)	7.02 % (3.36–12.99)

C & B: Culture and Biochemical test, CI: Confidence Interval (Binomial exact Calculation).

The geographical distribution of *Klebsiella* spp. varied among different upazilas of Sylhet district. The highest prevalence of *Klebsiella* spp. was noticed in Gowainghat (80.0 %), followed by Companiganj (77.78 %), while the lowest prevalence was observed in Bishwanath (20.83 %) and Jaintapur (23.53 %), as depicted in Fig. 2A. Notably, there was no detection of *K. oxytoca* in Gowainghat among the *Klebsiella* spp. positive isolates (Fig. 2C). Additionally, *K. oxytoca* was not detected in Osmaninagar, Jaintapur, and Bishwanath. In Kanaighat, the highest detection rates (18.75 %) of *K. oxytoca* were followed by Golabganj (14.28 %) among the positive *Klebsiella* spp. isolates. However, *K. pneumoniae* was found in every upazila in the Sylhet district, with the highest rates in Companiganj (55.56 %) and Fenchuganj (55.0 %), and the lowest in Bishwanath (12.5 %), as detailed in Fig. 2B.

### 3.2 *ESBLencodinggenefrequency*

**Fig. 3:**
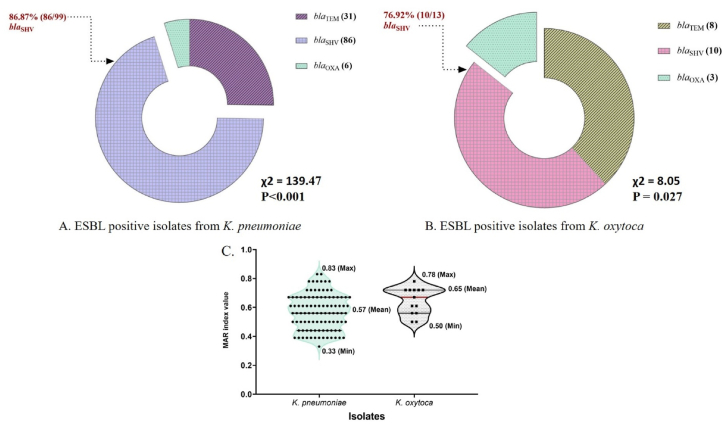
The map showing the ESBL resistant genes from the isolates of *K. pneumoniae* (Fig. A) and *K. oxytoca* (Fig. B). The violin plot indicates the multiple antibiotic resistance index (MARI) of *K. pneumoniae* and *K. oxytoca* isolated from poultry meat.

The positive isolates of *K. pneumoniae* (n = 99) and *K. oxytoca* (n = 13) were screened to detect ESBL encoding genes. The DDST method for phenotypic detection of ESBL depicted that 92.9 % (92/99) of *K.* *pneumoniae* isolates and 69.2 % (9/13) of *K.* *oxytoca* isolates tested positive. Genotypically, among the *K. pneumoniae* isolates, 86.87 % (86/99) harbored the *bla*_SHV_ gene, 31.3 % (31/99) carried the *bla*_TEM_ gene, and only 6.06 % (6/99) had the *bla*_OXA_ gene (Fig. 3A). Similarly, among the positive isolates of *K. oxytoca*, 76.92 % (10/13) harbored the *bla*_SHV_ gene, and only 23.08 % (3/13) carried the *bla*_OXA_ gene (Fig. 3B). No other ESBL encoding genes tested in this study were identified in the positive isolates.

### 3.3 *Antimicrobialresistantgenefrequency*

Among the positive isolates of *K. pneumoniae,* 95.96 % (95 out of 99) carried *tetA* gene, which is associated with tetracycline resistance. In addition, *sul1* genes were present in 81.82 % (81/99) of the ESBL-positive *K.* *pneumoniae* isolates. Of the four resistance genes analyzed, *aac(3)-iv* was found in only 7.07 % of the isolates. Similarly, positive isolates of *K. oxytoca* carried the *tetA* gene (92.31 %) and *sul1* genes (76.92 %) (Table 3).

**Table 3:** Genotypic resistance of commonly used antibiotics on retail poultry meat in Sylhet, Bangladesh.

Isolates (n)	Genotype	Results (+ve)	Percent % (95 % CI)	*Χ*^*2*^ value	*P* value
*K. pneumoniae* (99)				197.30	<0.001
	*tet(A)*	95	95.96 (89.98–98.89)		
	*str(A)*	69	69.70 (59.65–78.53)		
	*aac(3)-iv*	7	7.07 (2.89–14.03)		
	*Sul1*	81	81.82 (72.80–88.85)		
*K. oxytoca* (13)				16.75	<0.001
	*tet(A)*	12	92.31 (63.97–99.81)		
	*str(A)*	9	69.23 (38.57–90.91)		
	*aac(3)-iv*	2	15.38 (1.92–45.45)		
	*Sul1*	10	76.92 (46.19–94.96)		

n = No. of positive isolates, *X*^*2*^-goodness of fit test, *P* < 0.05 considered as level of significance. CI: Confidence Interval (Binomial exact test).

### 3.4 *Antibiogramandphenotype-genotypecorrelations*

**Fig. 4:**
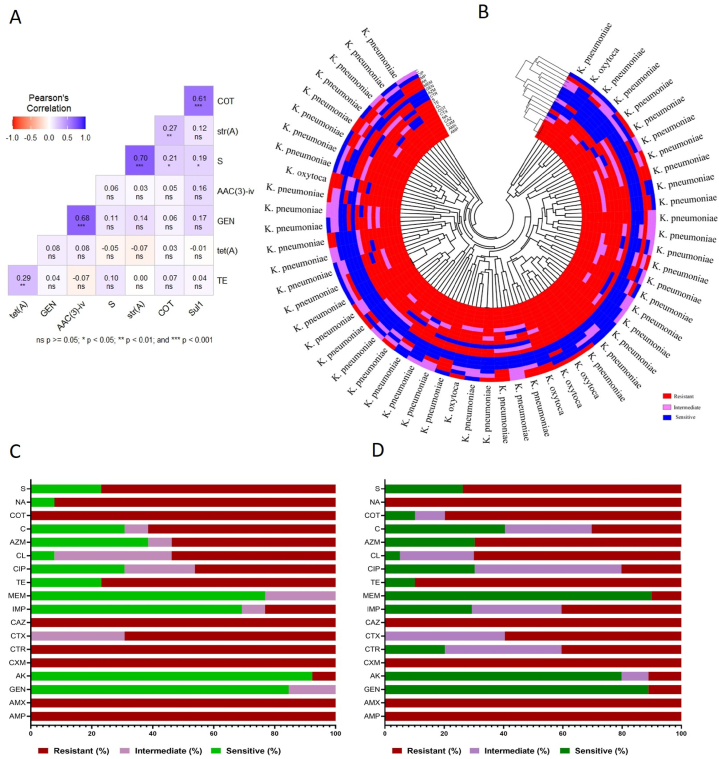
Fig. 4A showing the genotypic and phenotypic correlations on mostly used antimicrobials. Polar heatmap showing the resistant pattern of 18 antimicrobials (Fig. B). Antibiogram profile of *K. oxytoca* (Fig. C) and *K. pneumoniae* (Fig. D).

The antibiogram profile of *K. oxytoca* positive isolates (n = 13) indicated 100 % resistance to amoxicillin, ampicillin, ceftriaxone, cefuroxime, ceftazidime, and trimethoprim-sulfamethoxazole (Supplementary Table 1). The highest sensitivity was observed with amikacin (92.3 %) and gentamicin (84.6 %), followed by meropenem (76.9 %) and imipenem (69.2 %), as illustrated in Fig. 4B and C. Conversely, five antibiotics namely amoxicillin, ampicillin, cefuroxime, ceftazidime, and nalidixic acid showed 100 % resistance to the *K. pneumoniae* positive isolates (Supplementary Table 4). Meropenem (89.9 %) and gentamicin (88.89 %), followed by amikacin (79.8 %), demonstrated the highest sensitivity against *K. pneumoniae* (Fig. 4D).

Pearson's correlation analysis was conducted to assess the relationship between phenotypic resistance and the resistance genes of four commonly used antibiotics. The results are visualized in Fig. 4A. A moderately strong positive correlation (r = 0.68, *p < 0.001*) was identified between the presence of the *aac(3)-iv* gene and resistance to gentamicin. Similarly, streptomycin resistance exhibited a moderately strong positive correlation (r = 0.70, *p < 0.001*) with the presence of the *str(A)* gene. Additionally, a comparable positive correlation (r = 0.61) was observed between resistance to trimethoprim-sulphamethoxazole and the presence of the *sul1* gene. The correlation between the resistance phenotype and the corresponding resistance genes for tetracycline was weaker but still significant (r = 0.29, *p* < 0.01) which indicate that most isolates carry resistance genes that correlate with their phenotypic resistance patterns.

Interestingly, a positive correlation between streptomycin resistance genes *(strA)* and trimithoprim-sulphamethoxazole resistance was also found in this study, suggesting a potential linkage or co-selection mechanism between these resistance traits.

### 3.5 *MDR,XDR,PDRandMAR*

All positive isolates of *K.* *pneumoniae* (n = 99) and *K.* *oxytoca* (n = 13) were found to be resistant to multiple drugs, classifying them as MDR. However, no isolates were identified as XDR or PDR. The MAR index for *K. pneumoniae* ranged from 0.33 to 0.83, with an average of 0.57, while *K. oxytoca* isolates had a MAR index ranging from 0.50 to 0.78, with an average of 0.65 (Fig. 3C). These MAR index values suggest that the isolates were sourced from environments with high levels of resistance.

## 4 Discussion

*Klebsiella* spp. are opportunistic microorganism capable of colonizing both human and animal hosts, often identified as an incidental presence in commercially available meat products [39]. Identifying *Klebsiella* spp. in food resistant to antibiotic may empower public health authorities to devise more productive strategies for mitigating contamination. The current investigation revealed the actual prevalence of *Klebsiella* spp. in retail poultry meat and their antimicrobial resistance patterns.

In the present study, the actual prevalence of *Klebsiella* spp. was 57.89 % in Sylhet district, a rate similar to the findings from a study conducted in West Bengal, India (65 %) [40] and considerably exceeding rate reported in another study in Eastern China (13.8 %) [41]. Furthermore, studies in Shandong, China (98.88 %) and Ankara, Turkey (75.00 %) reported markedly higher prevalence of *Klebsiella* spp. compared to the current study's results [17,41]. The inconsistency may be attributable to the differing hygienic and sanitary standards applied during the slaughtering and retailing processes in different nations.

Among the *Klebsiella* spp. positive isolates, 83.19 % (99/119) samples were identified as *K. pneumoniae*, with a true prevalence of 41.75 % (95 % CI: 34.41–49.68). This prevalence is considerably higher than other studies which reported that the prevalence of *K. pneumoniae* in chicken meat samples was 21 % and 25.8 %, respectively [42,43]. Interestingly, the prevalence of *K. pneumoniae* in the current study closely aligns with findings from another study, which reported a prevalence of 47 % [44]. Additionally, the prevalence of *K. pneumoniae* was observed in another study which is significantly higher than the present study's findings, with the reported rate of 52.94 % [45]. In the case of *K. oxytoca*, the prevalence was 7.02 %, which is substantially lower than the prevalence reported in other studies, where the rates were 53 % and 27.4 %, respectively [44,46]. The variation in contamination rates between our study and others could be due to the different food sources analyzed in each investigation. Pinpointing the exact cause of the higher frequency of *K. pneumoniae* observed in our study is challenging, given the multitude of stages from farm production to consumer markets where infections might have originated. The geographical distribution of *Klebsiella* spp. in the Sylhet district exhibited considerable variation across different upazilas. The variations in prevalence could potentially be ascribed to factors such as improper management at retail outlets, the geographical setting of the shops in rural or urban areas, sanitary conditions, transportation methods, and the handling procedures utilized by the personnel.

Among the *K. pneumoniae* positive isolates, most samples 86.87 % (86/99) harbored *bla*_SHV_ gene which is somewhat consistent to the findings of a study for *bla*_SHV_ gene where prevalence was 66.67 % [47]. Similarly, 76.92 % (10/13) of the *K. oxytoca* isolates contained the *bla*_SHV_ gene, while 23.08 % (3/13) harbored the *bla*_OXA_ gene. No other ESBL-encoding genes were detected in these positive isolates. These findings are consistent with another study in poultry, which reported prevalence of 63.64 % for *bla*_TEM_, and 93.94 % for *bla*_SHV_ while 90.91 % for *bla*_OXA_ genes that is much higher than the present study [48]. However, a different study on poultry contradicts the present findings, reporting a 100 % prevalence of the *bla*_TEM_ gene [49]. Additionally, another study on cattle meat reported prevalence of 71.4 % for *bla*_SHV_ and a significantly higher prevalence of 72.2 % for *bla*_TEM_ genes [50]. In contrast, a study from Northwest South Africa found a nearly similar prevalence of the *bla*_TEM_ gene at 22.9 %, but a much lower prevalence of the *bla*_SHV_ gene at 34.3 % compared to our current findings [51]. In contrast, a different study reported a higher prevalence of the *bla*_TEM_ gene at 64.7 % and *bla*_SHV_ gene at 35.2 % [52]. In Korea, a study showed the highest percentages of the *bla*_TEM_ (100 %) and *bla*_SHV_ (94.1 %) genes in companion animals [53]. These prevalence disparities of ESBL genes could be linked with factors such as differences in antibiotic usage practices, management systems, and environmental conditions specific to each species. Additionally, variations in bacterial flora and the potential for interspecies transmission can influence the spread of these genes among different animal populations.

The present study demonstrated that the *tetA* and *sul1* genes exhibited the highest prevalence among the positive isolates of *K. pneumoniae* (95.96 % and 81.2 %) and *K. oxytoca* (92.31 % and 76.92 %). In contrast, the *aac(3)-iv* gene was identified in only 7.07 % of the isolates. These findings significantly exceed those of another study, which reported a prevalence of 32 % for *tetA* and 71 % for *sul1* [54]. Another study found that the prevalence of *tetA* and *sul1* exceeded 75 %, closely aligning with the present study's results [55]. Yet another study reported a prevalence of 72.58 % for *tetA*, 44.16 % for *sul1*, and 25.38 % for *aac(3)-iv*, which is consistent with the present study's findings for *tetA* and *sul1*, although the prevalence of *aac(3)-iv* was notably higher than in the present study [56]. Variations in the prevalence of antibiotic resistance genes in *Klebsiella* infections in poultry can arise from multiple factors, including geographic differences, antibiotic usage patterns, genetic diversity, hygiene and management practices, horizontal gene transfer mechanisms, and the methodologies employed for sampling and detection. These elements collectively influence the observed resistance patterns and highlight the complexity of addressing antibiotic resistance in agricultural settings.

The antibiogram profiles of *K. pneumoniae* and *K. oxytoca* isolates in our study revealed absolute resistance (100 %) to amoxicillin, ampicillin, ceftriaxone, cefuroxime, ceftazidime, nalidixic acid, and trimethoprim-sulfamethoxazole. These findings are closely aligned with those reported by Safika et al. [49], who observed resistance rates of 97.5 % for ampicillin and 95 % for nalidixic acid and trimethoprim-sulfamethoxazole. Furthermore, our results indicate substantially higher resistance rates compared to the study conducted by Fielding et al. [57]. Additional studies have also documented elevated resistance to nalidixic acid, tetracycline, sulfonamides, and trimethoprim-sulfamethoxazole, corroborating our findings [58]. Conversely, our study identified high susceptibility rates for gentamicin, amikacin, meropenem, and imipenem against *K. pneumoniae* and *K. oxytoca*. Our findings are also in close agreement with Gharavi et al. [59] who documented high susceptibility rates in *K. pneumoniae* for imipenem (99.3 %), amikacin (95.8 %), meropenem (90 %), and gentamicin (90 %), with notably lower susceptibility to ceftriaxone, cefuroxime, ceftazidime, tetracycline, and ampicillin. *K. oxytoca* isolates exhibited 100 % susceptibility to imipenem and 92.3 % susceptibility to amikacin, while displaying higher resistance to ampicillin ceftazidime, cefotaxime, norfloxacin, cefalexin, and co-trimoxazole.

The present study also demonstrated that all isolates of *K. pneumoniae* and *K. oxytoca* exhibited MDR, corroborating the results of a similar investigation where it was found that 53.57 % of *K. pneumoniae* isolates demonstrated MDR, a significantly lower proportion compared to the present study [60]. The findings of the present study are consistent with the study conducted in Shandong, China, which reported an MDR *K. pneumoniae* prevalence of 87.88 % [48]. Additionally, the present study's findings regarding MDR *K. oxytoca* closely aligned with another study that reported 90.97 % MDR *K. oxytoca* [61]. In contrast, other studies reported much lower MDR rates for *Klebsiella* spp., at 55 % [62,63]. Furthermore, the MAR index of *K. pneumoniae* in this study ranged from 0.33 to 0.83, which is consistent with findings from another study in Peshawar, Pakistan, where the MAR value of *K. pneumoniae* ranged from 0.21 to 0.92 [63]. Another study reported slightly higher results, with the MAR index of *K. pneumoniae* ranging from 0.2 to 1.0 [64]. Similarly, the positive isolates of *K. oxytoca* in the present study exhibited a MAR index spanned from 0.50 to 0.78, with an average of 0.65, which fully aligns with the findings of a study in Egypt where the MAR index of *K. oxytoca* was 0.636 [65].

In this study, we report a novel finding of *K. pneumoniae* and *K. oxytoca* carrying these types of MDR. We observed higher MDR patterns of the identified organisms and correlation with antimicrobial resistant genes. Our findings offer valuable data for tackling the spread of *bla*_TEM_ and *bla*_SHV_ producing *K. pneumoniae* and *K. oxytoca* in poultry meat in Sylhet and its potential transmission to humans.

This study focused solely on *K. pneumoniae* and *K. oxytoca*, common in the region, potentially overlooking other *Klebsiella* species. The absence of whole-genome sequencing (WGS) limits the comprehensive understanding of genetic diversity and resistance mechanisms. The research was also confined to a specific geographic area, which may limit the generalizability of the results to other regions with different ecological or farming conditions. Additionally, the study relied on cross-sectional data, which limits the ability to infer causal relationships. Seasonal variations were not accounted for, which may influence the prevalence and characteristics of the conditions studied. Addressing these limitations in future studies could provide more extensive insights and improve the overall applicability of the findings. Additionally, a larger sample size and longitudinal studies are recommended to monitor trends and assess the effectiveness of intervention strategies. Regular surveillance and improved hygiene practices are essential to control the spread of MDR *Klebsiella* spp. in poultry meat.

## 5 Conclusions

This study identifies retail poultry meat as a major reservoir of *Klebsiella* spp., including multidrug-resistant strains, highlighting significant public health concerns linked to foodborne transmission. The high prevalence of ESBL-producing isolates and associated resistance genes points to poultry as a critical vector for the spread of AMR in Bangladesh. These findings enhance our understanding of the role of the food chain in AMR dissemination and underscore the urgent need for robust food safety measures, systematic AMR monitoring, and effective regulatory frameworks in the poultry industry. Furthermore, the study emphasizes the critical importance of rational antibiotic use in both agricultural and clinical contexts to address the escalating global challenge of AMR.

## CRediT authorship contribution statement

**Fatema Yeasmin Tanni:** Writing – original draft, Software, Methodology, Investigation, Formal analysis, Data curation. **Md. Shahidur Rahman Chowdhury:** Writing – review & editing, Writing – original draft, Supervision, Project administration, Methodology, Investigation, Funding acquisition, Formal analysis, Data curation. **Hemayet Hossain:** Writing – review & editing, Writing – original draft, Software, Methodology, Investigation, Formal analysis, Data curation. **Md. Atik Faysal:** Methodology, Investigation, Data curation. **Md. Anisur Rahman:** Methodology, Investigation, Data curation. **Ahsan Al Emon:** Methodology, Investigation, Data curation. **Mashuka Nahida Asha:** Methodology, Investigation, Data curation. **Md. Mukter Hossain:** Writing – review & editing, Validation, Investigation, Formal analysis. **Md. Mahfujur Rahman:** Writing – review & editing, Writing – original draft, Visualization, Validation, Supervision, Software, Resources, Project administration, Methodology, Investigation, Funding acquisition, Formal analysis, Data curation, Conceptualization.

## Ethics declaration

• The work described has not been published previously in any format.
• The article is not under consideration for publication elsewhere.
• The article's publication is approved by all authors and by the Sylhet Agricultural University.
• If accepted, the article will not be published elsewhere in the same form, in English or in any other language, including electronically without the written consent of the copyright-holder.

## Data availability statement

The data supporting this study are available upon request from the corresponding author.

## Funding

This research was funded by the 10.13039/501100020392Sylhet Agricultural University Research System (10.13039/501100020392SAURES) with support from the University Grant Commission (10.13039/100015747UGC) of Bangladesh.

## Declaration of competing interest

The authors declare the following financial interests/personal relationships which may be considered as potential competing interests:Md. Mahfujur Rahman reports financial support was provided by 10.13039/501100020392Sylhet Agricultural University Research System (10.13039/501100020392SAURES). If there are other authors, they declare that they have no known competing financial interests or personal relationships that could have appeared to influence the work reported in this paper.
