# Supplementary material for: Prevalence and antimicrobial resistance of extended spectrum beta-lactamase (ESBL) producing Klebsiella spp. in poultry meat
Source: Heliyon. 2025 Jan 6;11(1):e41748. doi: 10.1016/j.heliyon.2025.e41748 (PMC11761286; doi:10.1016/j.heliyon.2025.e41748)
Supplement: Multimedia component 1 [file mmc1.docx]

**Supplementary Table 1: Thermal Cycle for mPCR of *Klebsiella* genus and *K. pneumoniae***

| **SL. No** | **Steps** | **Temperature (^0^ C)** | **Time** | **Cycle** | **Reference** |
| --- | --- | --- | --- | --- | --- |
| 1 | Initial Denaturation | 95^0^ C | 5 min | 1 | (Bobbadi et al., 2020) |
| 2 | Final Denaturation | 95^0^ C | 1 min | 35 |  |
| 3 | Annealing | 55^0^ C | 1 min |  |  |
| 4 | Initial Extension | 72^0^ C | 2 min |  |  |
| 5 | Final Extension | 72^0^ C | 10 min | 1 |  |

**Supplementary Table 2: Thermal Cycle for UniPCR of *K. oxytoca***

| **SL. No** | **Steps** | **Temperature (^0^ C)** | **Time** | **Cycle** | **Reference** |
| --- | --- | --- | --- | --- | --- |
| 1 | Initial Denaturation | 95^0^ C | 2 min | 1 | (Liza et al., 2024) |
| 2 | Final Denaturation | 94^0^ C | 20 sec | 35 |  |
| 3 | Annealing | 59^0^ C | 20 sec |  |  |
| 4 | Initial Extension | 72^0^ C | 30 sec |  |  |
| 5 | Final Extension | 72^0^ C | 10 min | 1 |  |

**Supplementary Table 3: Results of different biochemical tests of *Klebsiella* isolates from Poultry meat**

| **SL. No** | **Name of Test** | **Result** | **Interpretation** |
| --- | --- | --- | --- |
| 1 | Sugar fermentation Test by TSI Agar | Yellow color Slant and Butt | TSI + |
| 2 | Citrate Utilization test by Simmon’s Citrate Agar | Bright Blue color | Citrate + |
| 3 | MR Test | Yellow color | MR - |
| 4 | Motility, Indole, and Urease test by MIU agar | Motility absent | Motility - |
|  |  | Yellow-orange to pink-red | Urease + |
|  |  | No change of color | Indole – (*K. pneuminae*) |
|  |  | Pink color ring formation | Indole + (*K. oxytoca*) |

TSI = Triple Sugar Iron agar, MR = Methyl red test, MIU= Motility, Indole and Urease test

| Antibiotics | *K. pneumoniae* | | | *K. oxytoca* | | |
| --- | --- | --- | --- | --- | --- | --- |
|  | Sensitive | Intermediate | Resistant | Sensitive | Intermediate | Resistant |
| Ampicillin (AMP) | 0% | 0% | 100% | 0% | 0% | 100% |
| Amoxicillin (AMX) | 0% | 0% | 100% | 0% | 0% | 100% |
| Gentamicin (GEN) | 88.89% | 0% | 11.11% | 84.60% | 15.40% | 0% |
| Amikacin (AK) | 79.80% | 9.10% | 11.10% | 92.30% | 0% | 7.70% |
| Cefuroxime (CXM) | 0% | 0% | 100% | 0% | 0% | 100% |
| Ceftriaxone (CTR) | 20.20% | 39.39% | 40.40% | 0% | 0% | 100% |
| Cefotaxime (CTX) | 0% | 40.40% | 59.60% | 0% | 30.80% | 69.20% |
| Ceftazidime (CAZ) | 0% | 0% | 100% | 0% | 0% | 100% |
| Imipenem (IMP) | 29.30% | 30.30% | 40.40% | 69.20% | 7.70% | 23.10% |
| Meropenem (MEM) | 89.90% | 0% | 10.10% | 76.90% | 23.10% | 0% |
| Tetracycline (TE) | 10.10% | 0% | 89.90% | 23.10% | 0% | 79.90% |
| Ciprofloxacin (CIP) | 30.30% | 49.50% | 20.20% | 30.75% | 23.10% | 46.15% |
| Colistin (CL) | 5% | 25% | 69.70% | 7.69% | 38.50% | 53.90% |
| Azithromycin (AZM) | 30.30% | 0% | 69.70% | 38.50% | 7.70% | 53.90% |
| Chloramphenicol (C) | 40.40% | 29.30% | 30.30% | 30.80% | 7.70% | 61.50% |
| Trimethoprim-Sulfamethoxazole (COT) | 10.10% | 10.10% | 79.80% | 0% | 0% | 100% |
| Nalidixic Acid (NA) | 0% | 0% | 100% | 7.70% | 0% | 92.30% |
| Streptomycin (S) | 26.27% | 0% | 73.73% | 23.08% | 0 | 76.92% |

**Supplementary Table 4: Antibiogram profile of positive isolates of *K. pneumoniae* and *K. oxytoca***
